# Supplementary material for: Parental preferences for a mandatory vaccination scheme in England: A discrete choice experiment
Source: Lancet Reg Health Eur. 2022 Apr 13;16:100359. doi: 10.1016/j.lanepe.2022.100359 (PMC9097614; doi:10.1016/j.lanepe.2022.100359)
Supplement: Supplementary file 1 [file mmc1.docx]

**Captions for supplementary materials**

Supplementary materials 1. Selection of vaccine attributes

Supplementary materials 2. Full survey materials

Supplementary materials 3. Results of sensitivity analysis

Supplementary materials 4. Parental preferences for a mandatory vaccine scheme, sub-group analyses

Supplementary materials 5. Principal components analysis of psychological factors
